# Mental and Substance Use Disorders in Sub-Saharan Africa: Predictions of Epidemiological Changes and Mental Health Workforce Requirements for the Next 40 Years

**DOI:** 10.1371/journal.pone.0110208

**Published:** 2014-10-13

**Authors:** Fiona J. Charlson, Sandra Diminic, Crick Lund, Louisa Degenhardt, Harvey A. Whiteford

**Affiliations:** 1 School of Population Health, University of Queensland, Brisbane, Queensland, Australia; 2 Queensland Centre for Mental Health Research, Brisbane, Queensland, Australia; 3 Institute of Health Metrics and Evaluation, University of Washington, Seattle, Washington, United States of America; 4 Alan J Flisher Centre for Public Mental Health, Department of Psychiatry and Mental Health, University of Cape Town, Cape Town, South Africa; 5 National Drug and Alcohol Research Centre, University of New South Wales, Sydney, New South Wales, Australia; 6 Melbourne School of Population and Global Health, University of Melbourne, Melbourne, Victoria, Australia; Peking University, China

## Abstract

The world is undergoing a rapid health transition, with an ageing population and disease burden increasingly defined by disability. In Sub-Saharan Africa the next 40 years are predicted to see reduced mortality, signalling a surge in the impact of chronic diseases. We modelled these epidemiological changes and associated mental health workforce requirements. Years lived with a disability (YLD) predictions for mental and substance use disorders for each decade from 2010 to 2050 for four Sub-Saharan African regions were calculated using Global Burden of Disease 2010 study (GBD 2010) data and UN population forecasts. Predicted mental health workforce requirements for 2010 and 2050, by region and for selected countries, were modelled using GBD 2010 prevalence estimates and recommended packages of care and staffing ratios for low- and middle-income countries, and compared to current staffing from the WHO Mental Health Atlas. Significant population growth and ageing will result in an estimated 130% increase in the burden of mental and substance use disorders in Sub-Saharan Africa by 2050, to 45 million YLDs. As a result, the required mental health workforce will increase by 216,600 full time equivalent staff from 2010 to 2050, and far more compared to the existing workforce. The growth in mental and substance use disorders by 2050 is likely to significantly affect health and productivity in Sub-Saharan Africa. To reduce this burden, packages of care for key mental disorders should be provided through increasing the mental health workforce towards targets outlined in this paper. This requires a shift from current practice in most African countries, involving substantial investment in the training of primary care practitioners, supported by district based mental health specialist teams using a task sharing model that mobilises local community resources, with the expansion of inpatient psychiatric units based in district and regional general hospitals.

## Introduction

The recent Global Burden of Disease Study (GBD 2010) is the largest systematic assessment of disease and injury-specific epidemiology undertaken since its 1990 predecessor [1]. The GBD methodology incorporates the years of life lost through premature mortality (YLL) and years lived with disability (YLD) into a single metric (the Disability Adjusted Life Year (DALY)). Calculating disease burden worldwide and for 21 regions for 1990, 2005, and 2010 with methods to enable meaningful comparisons over time, it has delivered much needed information and key global health messages. The most notable of these is that the world has undergone a rapid health transition over the 1990 to 2010 period – populations have aged, infectious and childhood diseases are making way for non-communicable and chronic disorders, and disease burden is increasingly defined by disability rather than premature death [2].

The health transitions observed globally in GBD 2010 were not as apparent across Sub-Saharan Africa (SSA). Improvements in life-expectancies have trailed other regions, largely due to the HIV/AIDS epidemic, maternal deaths, and child mortality caused by infectious diseases and malnutrition. However these trends are at a turning point and the next 40 years are predicted to see significant reductions in child mortality and lowering mortality from HIV/AIDS and malaria, signalling the inevitable health transitions we have seen across the rest of the globe and a surge in the impact of chronic and non-communicable diseases, defined by long-term disability [3]–[6].

Mental and substance use disorders are the leading cause of disability, accounting for 23% of all disability-associated burden (years lived with a disability, YLD) globally and 19% in Sub-Saharan Africa in 2010 [7], [8]. Major depressive disorder (MDD) makes the largest contribution, accounting for approximately 40% of YLDs in this group, yet the very limited mental health services in Sub-Saharan Africa are frequently restricted to tertiary psychiatric facilities treating those with acute psychoses, and to humanitarian responses to traumatic events such as gender-based violence and societal conflict [9].

For most, the idea of a non-communicable disease epidemic in Sub-Saharan Africa may seem intangible, or at the very least, too far off to contemplate – particularly given the fact that communicable, maternal, neonatal and nutritional diseases still account for approximately 68% of DALYs in the region [7]. So, how imminent is this health transition, and what might it look like for Sub-Saharan Africa? Using UN population data [10] and the findings of GBD 2010, we estimate the change in burden of mental and substance use disorders in context of the broader epidemiological transition which will take place in Sub-Saharan Africa from 2010 to 2050, and model the predicted increase in the mental health workforce required to meet future needs.

## Methods

### GBD 2010

As part of the GBD 2010 study [4], burden of disease estimates were produced for 20 mental and substance use disorders [8]. In brief, this process consisted of systematic reviews for empirical epidemiological data for each disorder as described by the Diagnostic and Statistical Manual of Mental Disorders (DSM) and/or the International Classification of Diseases (ICD) [11], [12]. Internally consistent epidemiological models were created for each disorder using DisMod-MR, a Bayesian meta-regression tool. Adjustments to the data were made via various methods including the application of covariates and severity distributions, and comorbidity adjustments were applied to sequela-specific disability weights in the calculation of YLDs (more detailed information can be found in Murray et al 2012 [4] and Whiteford et al 2013 [8]).

For the GBD 2010, YLDs per person from a sequela (e.g. severe major depression) are equal to the prevalence of the sequela multiplied by the disability weight for the health state associated with that sequela. YLDs for a disease or injury are the sum of the YLDs for each sequela (e.g., mild, moderate and severe depression) associated with the disease or injury [13].

### Disability (YLD) estimates 2010 to 2050

YLD predictions for each 10-year period from 2010 to 2050 were calculated for the East, West, Central and Southern Sub-Saharan African regions (as defined by the Global Burden of Disease Study; refer to appendix S1 for a list of countries and regions). We applied UN population data forecasts (see appendix S1 for UN population data) to 2010 age-specific YLD rates ascertained from the GBD 2010 to calculate changes in YLDs for mental and substance use disorders in the context of communicable (includes communicable, maternal, neonatal and nutritional diseases) and non-communicable diseases for each Sub-Saharan region and 10-year time period (see appendix S1 for full list of mental and substance use disorders). By using 2010 YLD rates we assume that age-specific prevalence and disability weights will remain constant throughout the time period. It has been shown that prevalence and disability weights are unlikely to change significantly over time for the majority of mental disorders; however, there will likely be changes for substance use disorders and the implications of this assumption are addressed in the discussion [8].

### Service requirements for 2010 and 2050

Recommended packages of care and service requirements in low- and middle-income countries[14]–[16] formed the basis of estimation methods of full time equivalent (FTE) staffing requirements. Guided by the priority disorders identified in Bruckner et al [14], 7 mental and substance use disorders which were included in GBD 2010 were selected for modelling. Selected disorders were schizophrenia, bipolar disorder, depression, alcohol dependence disorder, opioid dependence disorder, conduct disorder and attention deficit hyperactivity disorder (ADHD) (the latter two for 5–19 year olds only). The seven disorders selected are not intended to represent the only important conditions (dementia and epilepsy are examples of other important conditions that could be added to future modelling).

### Prevalence estimates for 2010 and 2050

Disorder prevalence estimates are central to the estimation of FTE staff requirements. The more recent prevalence estimates from GBD 2010 were used over the prevalence estimates reported in Bruckner et al. Pooled prevalence estimates for each disorder and each of four Sub-Saharan Africa regions (East, West, Southern and Central) were calculated using 2010 age and sex-standardised prevalence estimates derived from DisMod-MR modelling. Predicted pooled prevalence for 2050 was also calculated using 2010 age and sex-standardised prevalence estimates and UN population data for 2050 (see appendix S1 for UN population figures). Pooled prevalence for the paediatric disorders (ADHD and conduct disorder) were based on only prevalence estimates from the 5–19 year age groups. Using similar methods, pooled prevalence estimates for the entire Sub-Saharan Africa (4 regions) were also calculated. Prevalence estimates at the regional level were applied to each country within that region.

Adjustments to prevalence estimates were necessary to account for comorbidity between disorders. To allow for comorbidity between depression, alcohol and opioid use disorders, the separate and combined prevalence of MDD, alcohol use, alcohol dependence, drug use and drug dependence were sourced from South African survey data published by Williams et al [17]. The GBD prevalence estimates for depression, alcohol and opioid use disorders were reduced in proportion to their weighting relative to the prevalence of any depressive or substance use disorder. This was translated into the formula: (disorder prevalence divided by sum of prevalences of individual depressive and substance use disorders) multiplied by (prevalence of any depressive or substance use disorder). Across the board, rates were adjusted down to 87.6% of the original total. This method was based on Lund et al [18].

It was assumed that conduct disorder and ADHD held no comorbidity with other disorders. However it was considered implausible to assume no comorbidity in the model between conduct disorder and ADHD. Data from the US National Comorbidity Survey Replication – Adolescent [19] were therefore used to estimate comorbidity between these childhood disorders, as no African data were available. As per the method for depressive and substance use disorders above, the raw prevalence of ADHD, conduct and oppositional defiant disorders, as well as the prevalence of any of these behavioural disorders, was used to calculate a comorbidity weighting (prevalence/sum of individual behavioural disorders) x (prevalence of any behavioural disorder). The GBD prevalence rates for ADHD and conduct disorder were adjusted down to 69.8% of the originals. A table summarising the pooled prevalence of selected mental and substance use disorders in Sub-Saharan Africa from GBD 2010, by region and adjusted for comorbidity can be found in the appendix S1.

For the purpose of the modelling (and due to a lack of data to estimate otherwise) it was assumed that bipolar disorder and schizophrenia held no significant comorbidity with any other mental disorders. Although these two disorders experience high comorbidity with substance use disorders, it was considered that the treatment patients would require for their substance use disorder would be in addition to that for their bipolar disorder or schizophrenia and therefore comorbidity adjustments were not made. The implications of this are explored in the discussion.

### Treatment packages

Treatment coverage targets and care packages (service coverage and utilization rate for each type of service) for the priority disorders were taken from Bruckner et al [14] (see appendix S1 for more details). A slight modification to the Bruckner et al model was made where an equivalent psychosocial treatment package as that for alcohol use disorders was added for opioid use disorders in line with WHO opioid treatment guidelines [20].

For each disorder, region or country and service type, the number of bed-days for inpatient or residential or number of sessions for outpatient care was equal to the *population X adjusted prevalence X treatment coverage target X service coverage X utilisation rate,* where: *treatment coverage target* is the percentage of prevalent cases requiring or presenting for any treatment; *service coverage* is the percentage of treated cases needing care in this specific service setting in a year; and *utilisation rate* is the average number of bed days used per case per year for cases treated in inpatient services, or the average number of consultations or sessions per case per year for cases treated in outpatient services.

### Staffing ratios

Staffing ratios recommended by the mhGAP costing tool [16] allowed estimation of staffing by health professional type – psychiatrist, other physician/doctor, nurse, psychologist, other psychosocial worker, and other provider/worker. The overall ‘nurse’ category was further split into psychiatric nurses and general nurses using a ratio of 1∶4, based on consultation with African mental health experts in four countries (further field testing is needed to validate this in future, as the figure will vary by country and setting).

### FTE staffing estimates by service type

FTE modelling was conducted for both 2010 and 2050, for each of the four Sub-Saharan African regions and a small selection of countries within each region. The selection of countries was done without prejudice and merely as examples to highlight the stark differences that can be observed across countries within the same region. The countries selected for modelling FTE were: Sub-Saharan Africa East – Zambia, Ethiopia, Burundi; Sub-Saharan Africa West – Ghana, Nigeria, Chad; Sub-Saharan Africa Central – Democratic Republic of Congo (DRC), Angola; Sub-Saharan Africa Southern – South Africa, Zimbabwe.

For each region/country, the number of bed-days for inpatient/residential services and sessions for outpatient services were converted into FTE mental health staff requirements using the following formulae. The number of FTE staff required to deliver inpatient/residential mental health care was calculated as: number of bed-days/0.85 (85% bed occupancy rate as per Bruckner et al)/365 service days per annum/25 beds in unit x number of staff per unit (7.5 per 25 beds as per mhGAP) x staffing ratio (proportion of nurses, doctors, and other professionals in that setting from mhGAP).

The number of FTE staff required to deliver outpatient mental health care was calculated as: number of sessions/number of consultations per day/240 working days per year (from mhGAP) X staffing ratio (from mhGAP) [14],[21]. Consultations per day were derived from mhGAP as a weighted average across providers in each category of outpatient care: outpatient care −8 consultations per day; primary care −9 consultations per day; psychosocial (ancillary) care −7 consultations per day. Group day programs were assumed to be delivered to an average of 10 users per day.

Finally, gaps between the estimated target FTE staff for 2010 and the current mental health service FTE staffing levels in the selected countries, as reported by the WHO Mental Health Atlas [9], were estimated.

## Results

### Burden of disease

**Figure 1:**
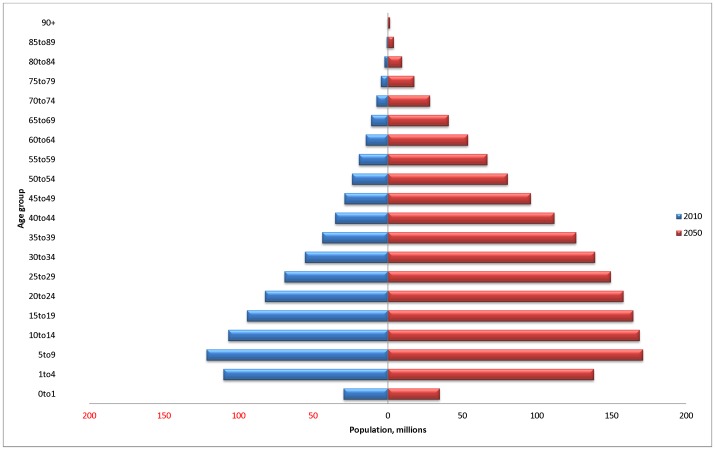
Population age distribution in Sub-Saharan Africa, 2010 and 2050. Source: United Nations Population Data [36].

The population distribution in Sub-Saharan Africa is expected to change dramatically over the next 40 years (Figure 1). An expected doubling of population size from approximately 0.9 billion to 1.8 billion will be accompanied by a significant ageing. As a result, a population dominated by under 25 s in 2010 (63%) will be represented by over 25 s in 2050 (53%).

**Figure 2:**
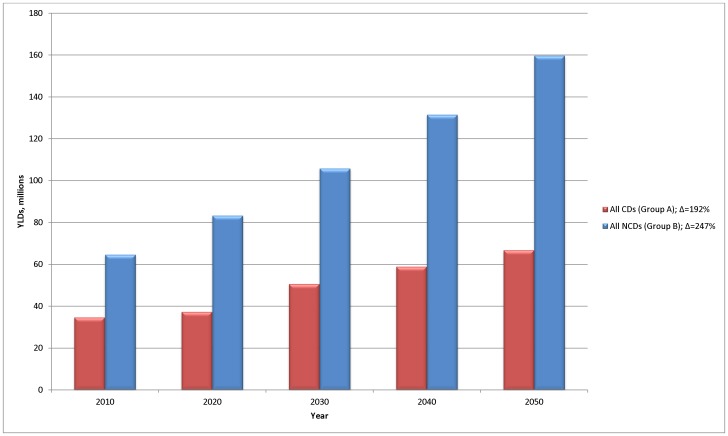
Change in disability burden distribution of communicable and non-communicable diseases in Sub-Saharan Africa. Note: CDs include all communicable, maternal, neonatal and nutritional diseases; NCDs include all non-communicable diseases (including mental and substance use disorders).

This change in demographics will result in a rapidly growing disparity between the disability burden associated with communicable compared to non-communicable diseases, with a one and a half fold increase in non-communicable compared to communicable diseases (Figure 2). Interestingly, although considering only population ageing and growth would see communicable diseases experience a doubling in burden, the burden in under 5 s – traditionally the most important group for communicable diseases – could see only a relatively modest increase over time of around 24% (appendix S1).

**Figure 3:**
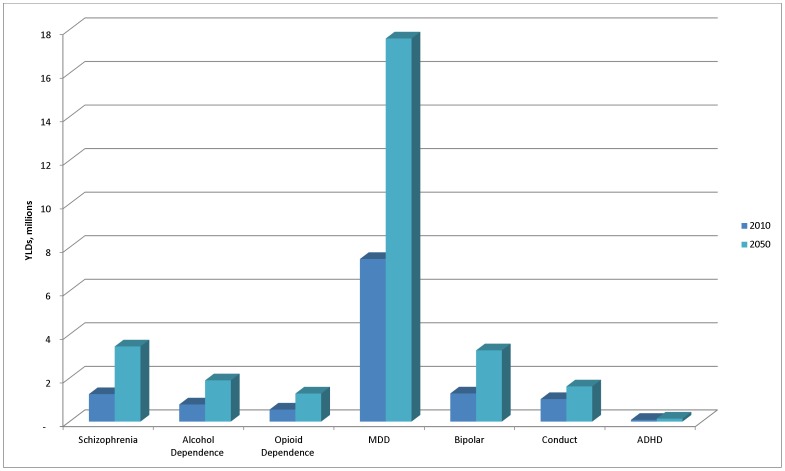
Disability burden for individual mental and substance use disorders, 2010 to 2050.

In terms of individual disorders, the largest contributor to burden of disease is MDD (Figure 3). This is followed by schizophrenia which is considered to be one of the most disabling conditions across all diseases in GBD. The childhood disorders of conduct disorder and ADHD will see relatively little increase in burden as the populations of SSA ages significantly over the next 40 years.

**Figure 4:**
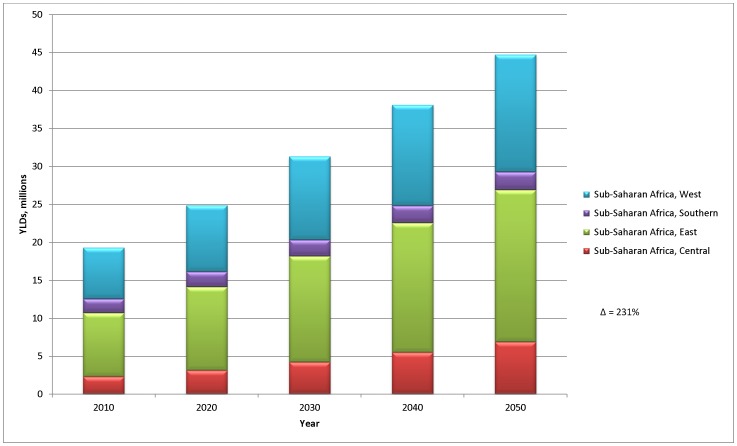
Disability burden of mental and substance use disorders in Sub-Saharan Africa over time, all ages.

In terms of mental and substance use disorders, all Sub-Saharan African regions would experience an increase in burden of around 130% in the absence of a change in disorder prevalence rates (Figure 4). This increase would vary across regions, with the largest (196%) seen in Sub-Saharan Africa Central and the lowest (28%) in Sub-Saharan Africa Southern (East = 139% and West = 129%).

Mental and substance use disorders were the leading cause of YLDs in Sub-Saharan Africa in 2010 (18.94% of total) and an estimated rise from approximately 20 million YLDs to 45 million YLDs could be experienced by 2050. Based on the individual effects of demographic changes alone, by 2050 mental and substance use disorders may be equivalent to approximately two thirds the YLDs of the entire communicable diseases group (67 million YLDs, appendix S1).

**Figure 5:**
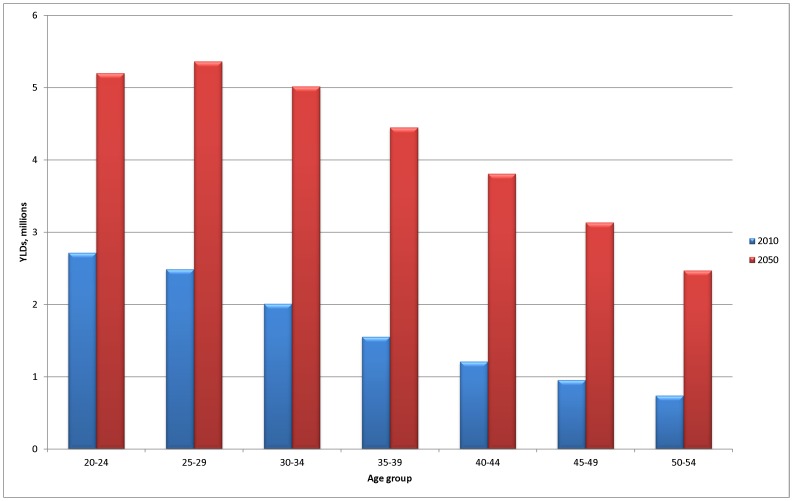
Change in disability burden of mental and substance use disorders over time, 20–54 years by age group, all Sub-Saharan Africa.

Importantly, the shifting population distribution of Sub-Saharan Africa seen in Figure 1 translates directly to the mental and substance use burden changes seen in Figure 5, which shows the increase in burden by age group. This is important for this group of disorders, where the 20–54 age group is typically most affected.

### Health service requirements

The overall predicted increase in target FTE per 100,000 population in each region from 2010 to 2050 is 24.0 to 24.3, due to demographic changes influencing overall prevalence (Table 1). This seemingly modest increase can be misleading though and only when converted to absolute FTE do the estimates become more meaningful in terms of health system planning. Target FTE for Sub-Saharan Africa Southern in 2050 will increase by around 3,700 from the 2010 target, whilst the target FTE for Sub-Saharan Africa East, a far more populous region, will need to increase by 95,400 over the 2010 to 2050 time period to meet the growth in burden of disease.

**Table 1:** Mental health workforce requirements for Sub-Saharan Africa, year 2010 and 2050^1^^,^ 2.

	Sub-Saharan Africa, East			Sub-Saharan Africa, West			Sub-Saharan Africa, Central			Sub-Saharan Africa, Southern		
	Target FTE 2010	Target FTE 2050	Suggested increase^3^	Target FTE 2010	Target FTE 2050	Suggested increase	Target FTE 2010	Target FTE 2050	Suggested increase^3^	Target FTE 2010	Target FTE 2050	Suggested increase^3^
*Provider type*												
Psychiatrist	1.8	1.9	7,400	1.7	1.8	6,200	1.9	1.9	2,900	1.8	1.9	300
Medical officer/clinical officer	2.5	2.5	9,900	2.3	2.4	8,200	2.5	2.6	4,000	2.5	2.5	400
Psychiatric nurse	2.1	2.2	8,500	2.0	2.0	7,100	2.2	2.2	3,400	2.1	2.2	300
General nurse	8.5	8.6	33,800	8.1	8.2	28,200	8.6	8.8	13,500	8.6	8.7	1,300
Psychologist	2.0	2.1	8,100	1.9	2.0	6,800	2.1	2.1	3,200	2.0	2.1	300
Psychosocial provider	3.5	3.6	14,100	3.3	3.4	11,700	3.6	3.7	5,600	3.5	3.6	500
Other primary care	3.5	3.5	13,800	3.2	3.3	11,200	3.5	3.6	5,500	3.5	3.5	500
*Service type*												
Outpatient care	10.9	11.0	42,800	10.2	10.2	34,900	11.1	11.3	17,200	11.0	11.1	1,600
Psychosocial care	2.5	2.6	10,400	2.2	2.3	8,100	2.6	2.7	4,200	2.6	2.6	400
Inpatient care	10.5	10.7	42,300	10.2	10.4	36,400	10.6	10.9	16,700	10.6	10.9	1,700
Total	24.0	24.3	95,400	22.6	22.9	79,400	24.4	24.8	38,100	24.2	24.6	3,700

1 FTE are expressed as a rate per 100,000 population.

2 The suggested increase to 2050 was calculated by subtracting the target full time equivalent for 2050 in absolute numbers from that of 2010. ^3^Rounded to nearest hundred.

**Figure 6:**
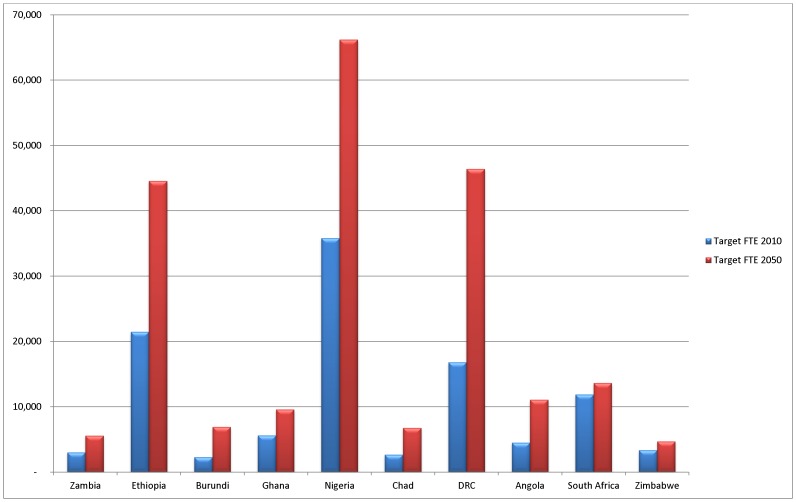
Predicted increase in FTE staff requirements for mental health care for selected Sub-Saharan African countries, 2010 to 2050. Note: SSA East – Zambia, Ethiopia, Burundi; SSA West – Ghana, Nigeria, Chad; SSA Central – DRC, Angola; SSA Southern – South Africa, Zimbabwe.

Not only are there significant differences in target FTE increases across the 4 regions, but also within the countries of each region (Figure 6). The highly populous countries of Ethiopia, Nigeria and DRC highlight well the impact of population growth and ageing on health system requirements when compared against their neighbouring countries. Nigeria is predicted to require an additional 65,000 FTE staff to provide mental health care by 2050. In contrast, increases required in South Africa and Zimbabwe are expected to be much smaller.

Perhaps more important than predicted increases in target FTE is the gap between the estimated minimum FTE staffing requirements for 2010 and the actual FTE staffing numbers that currently exist within countries as reported by the WHO Mental Health Atlas. The health challenges in Sub-Saharan Africa are clearly demonstrated here with some countries reportedly having only a fraction of current target staffing levels. Although current FTE estimates from the WHO Mental Health Atlas and FTE targets proposed in this paper are not strictly comparable across the board, two groups of mental health workers, psychiatrists and psychologists, can provide a reasonable indication of any shortfall in mental health workers within a country (Table 2). Our estimates highlight a very significant shortfall of mental health workers across all SSA countries, with large variations within the same geographical regions. Even South Africa, with clearly the best mental health staffing capacity, appears to be lacking in crucial areas such as numbers of psychiatrists. Particularly notable is Nigeria, a country with low current capacity and one of the highest predicted increases in requirements.

**Table 2:** Current and target 2010 FTE psychiatrists and psychologists per 100,000 population for selected countries in Sub-Saharan Africa.

Country	Psychiatrists			Psychologists		
	Current	2010 target	% target FTE	Current	2010 target	% target FTE
*Sub-Saharan Africa East*						
Zambia	0.03	0.8	4%	0.02	0.3	7%
Ethiopia	0.04	0.8	5%	0.02	0.3	7%
Burundi	0.01	0.8	1%	0.01	0.3	3%
*Sub-Saharan Africa West*						
Ghana	0.07	0.8	9%	0.04	0.3	13%
Nigeria	0.06	0.8	8%	0.02	0.3	7%
Chad	0.01	0.8	1%	0.01	0.3	3%
*Sub-Saharan Africa Central*						
DRC	0.07	0.8	8%	0.015	0.3	5%
Angola	0.002	0.8	0.3%	0.003	0.3	1%
*Sub-Saharan Africa Southern*						
South Africa	0.27	0.8	34%	0.31	0.3	103%
Zimbabwe	0.06	0.8	8%	0.04	0.3	13%

## Discussion

We used UN population data to forecast the change in disability-associated burden of seven of the major mental and substance use disorders in Sub-Saharan Africa from 2010 to 2050. It is estimated that during this 40 year period the population will double in size and age significantly, making way for the dramatic health transition seen in other regions of the world. Predictions clearly highlight the anticipated shift from communicable diseases characterised by high mortality to non-communicable diseases with a chronic and disabling course.

Naturally, modelling burden estimates holding YLD rates constant makes several assumptions, including that disorder prevalence and disability weights remain constant. Whilst this is entirely plausible for many non-communicable diseases, indeed the prevalence of many mental disorders has been shown to not change significantly over time [8], this may not be true for others. Drug use disorder prevalence, for example, is expected to increase in the Sub-Saharan Africa regions in the coming years, largely driven by changing routes of drug trafficking [22], [23].

Modelling changes in communicable diseases is also not straight forward. Different factors are at play and will influence the estimates. Firstly, the successes of public health campaigns such as expanded programs on immunisation and insecticide treated nets to combat malaria should continue to reduce the prevalence of infectious diseases. Secondly, improvements in health systems and treatments may not reduce the prevalence of some communicable diseases but shift the burden from premature mortality to a more chronic health loss; the push towards universal access to anti-retroviral therapies makes HIV the definitive example of this and will shape the future burden of disease in Sub-Saharan Africa substantially. In terms of disability, it is possible that the gains made by declines in prevalence of communicable, maternal, neonatal and nutritional diseases over the next 40 years will be partially offset by the increase in burden of chronic HIV. Whatever the scenario for communicable disease may be, it is clear that the balance in the contribution of communicable and non-communicable diseases to disease burden is set to change dramatically.

The consequences of a rising burden on non-communicable disease (NCD) are far-reaching. For the mental and substance use disorders, largest group of NCD’s the impacts are long-lasting at the level of the individual, family and community [24]. Quality of life is impacted and economic costs are significant. A recent study estimates that the cumulative global impact of mental disorders may amount to US$16 trillion of lost economic output over the next 20 years, equivalent to 25% of global GDP in 2010 [25].

The secondary health outcomes of mental disorders also need to be considered. For example, major depression has been shown to be an independent risk factor for other important non-communicable disorders such as ischaemic heart disease [26] and a consistent association between HIV and poor mental health has been reported [27]. The strong and often bidirectional relationships that exist between mental and substance use disorders with communicable disease, non-communicable disease and injuries emphasises the critical role of mental health [27].

Due to the pressures of communicable disease and malnutrition, mental health in Africa has been low on the priority list to-date. In terms of policy, a recent WHO report has revealed only 42.2% of countries within the WHO Africa region report having a dedicated mental health policy, 67% possess a mental health plan and 44% report having dedicated mental health legislation [9]. African countries reportedly spend less than 1% of their health budgets on mental health [28].

As a result mental health services are poorly resourced and generally accessible to only the most severely ill and then as inpatients in urban facilities. Mental health care provided outside the hospital by health and social workers based in the community is only available in half of African countries [28]. Globally, treatment rates for people with mental and substance use disorders remain low [29], [30], with treatment gaps over 90% in low and middle income countries [29]. Perhaps an exception to this is in traumatised populations where non-government organisations are present, providing temporary mental health services during emergency or post-conflict response efforts. However, these services are not sustainable and it is important to prepare for chronic disease and disability services beyond acute care.

In keeping with the recommendations of WHO, reaching FTE targets set in this paper requires a shift from current practice in most African countries, where psychiatric hospitals are the main site of service delivery, and consume the vast majority of the country’s mental health budget [9]. Instead a new model is required which involves substantial investment in the training of primary care practitioners, supported by district based mental health specialist teams, using a task sharing model that mobilises local community resources [31], [32]. The new model also requires substantial investment in smaller inpatient psychiatric units, based in district and regional general hospitals as reflected in the inpatient services targets which demand the biggest increase in resources in this model.

Health transitions in other regions of the world have been rapid [4]. The health system reforms required to deal with dramatic changes in burden of disease are intimidating and will take long term careful planning. However the human and economic costs of not preparing for this will be substantial. Sub-Saharan Africa is in a particularly challenging position as it will soon be caught in the rising tide of non-communicable disease, yet it still has a considerable way to go in the fight against communicable, maternal, neonatal and nutritional diseases. A logical starting point in the preparation might be to implement packages of care for key mental disorders which have been designed for low- and middle-income countries and provide realistic goals given challenging environments such as those in Sub-Saharan Africa [21], [33]. Fundamental in scaling up services is increasing the mental health workforce [14] and integrating mental health into primary health care [34], [35]. A continuing commitment to addressing key research priorities is also vital [34].

### Limitations

As highlighted previously, this modelling is limited to disability-associated burden only and makes the assumption of unchanged disease prevalence and disability weights over time. Whilst this may be a reasonable representation for the future of many non-communicable diseases, changes in disease profiles of communicable diseases will be likely. As mental and substance use disorders are highly disabling conditions which carry a relatively small amount of directly-associated mortality and there will be a future shift to chronic, disabling diseases it was seen appropriate to selectively present disability-associated burden forecasts.

One obvious omission from this paper which is particularly relevant to the Sub-Saharan African context is that of neurological disorders. Neurological disorders were modelled separately from mental and substance use disorders in GBD 2010 and were not included in our modelling. Given epidemiological transition and ageing population, the inclusion of neurological disorders (dementia in particular) would add substantially to the predicted changes in burden and FTE requirements and have significant implications for concomitant service requirements.

Other assumptions of our estimates are that predicted demographic changes are correct. The estimation process of mental disorder YLD rates ascertained in GBD 2010 additionally carried some limitations which have been discussed elsewhere [8]. In particular, the lack of data from the Sub-Saharan region was problematic and more epidemiological data is required from these regions.

Comorbidity adjustments were not made between bipolar disorder and schizophrenia and substance use disorders when the known comorbidity is high. There was no clear way to adjust prevalence between these disorders in a way that would reflect the reality of how service requirements would likely reduce (that is, a reduction in substance use services) given the clinical reality that any reduction in service requirements for a patient experiencing bipolar disorder or schizophrenia and a substance use disorder would be minimal given the very different packages of care, intensity of treatment required and the low prevalence of these two mental disorders. Although there is potential that treatment services for substance use disorders have been overestimated to a small degree, it should be noted that estimated staffing requirements for the treatment of opioid and alcohol use disorders will likely be conservative due to using dependence (lower) prevalence estimates, as provided by GBD 2010, with treatment targets and care packages from Bruckner et al designed for the broader category of abuse and dependence.

It is important to recognise that the model used for estimating service requirements in this paper is one developed for both low- and middle-income countries and may need substantial modification to suit the Sub-Saharan African context. Furthermore, it is recognised that each country will have its own health service and workforce configuration and unique challenges, and therefore targets should ideally be tailored to the country context.

Finally, the Mental Health Atlas only records mental health labelled staff working in dedicated facilities/clinics whereas the care packages presented by Bruckner and colleagues represent the total package of care that person needs, including care that might be provided by non-mental health specialists working in general primary care clinics. These key differences make general comparisons against the staffing targets modelled above difficult yet the data is nonetheless informative.

## Conclusion

With a rapid epidemiological transition predicted for Sub-Saharan African over the coming decades it is now time to begin planning for health services able to address a dramatic increase in burden of non-communicable disease. Mental and substance use disorders are already the leading cause of disability in Sub-Saharan Africa yet services are drastically under-resourced. Packages of care and service models advocating for a task shifting approach and the embedding of mental health services within the primary health care system are available for LMICs. Investment and health system reform should be a priority for policy makers in Sub-Saharan Africa.

## Supporting Information

Appendix S1 (PDF)
