# Supplementary material for: Mental and Substance Use Disorders in Sub-Saharan Africa: Predictions of Epidemiological Changes and Mental Health Workforce Requirements for the Next 40 Years
Source: PLoS One. 2014 Oct 13;9(10):e110208. doi: 10.1371/journal.pone.0110208 (PMC4195720; doi:10.1371/journal.pone.0110208)
Supplement: Appendix S1 — (PDF) [file pone.0110208.s001.pdf]

Supplementary Information

Table S1: List of GBD Sub-Saharan Africa regions and countries[1]

Region	Countries
Sub-Saharan Africa, Central	Angola, Central African Republic, Congo, Democratic Republic of the Congo, Equatorial Guinea, Gabon
Sub-Saharan Africa, East	Burundi, Comoros, Djibouti, Eritrea, Ethiopia, Kenya, Madagascar, Malawi, Mozambique, Rwanda, Somalia, South Sudan, Sudan, Tanzania, Uganda, Zambia
Sub-Saharan Africa, Southern	Botswana, Lesotho, Namibia, South Africa, Swaziland, Zimbabwe
Sub-Saharan Africa, West	Benin, Burkina Faso, Cameroon, Cape Verde, Chad, Côte d'Ivoire, Ghana, Guinea, Guinea-Bissau, Liberia, Mali, Mauritania, Niger, Nigeria, Sao Tome and Principe, Senegal, Sierra Leone, The Gambia, Togo

Table S2: Population data: 2010 and 2050 UN population by region

Region	Total population		Population 5-19 years	
	2010	2050	2010	2050
SSA Central	98,041,204	249,836,218	37,549,425	81,588,522
SSA East	357,361,615	744,241,063	135,303,449	212,183,073
SSA Southern	70,352,580	84,161,080	22,987,773	20,136,630
SSA West	338,971,179	679,859,364	127,245,433	190,106,084
Total SSA	864,726,578	1,758,097,725	323,086,080	504,014,309

Table S3: List of GBD mental and substance use disorders[1]

**Mental disorders**	**Drug use disorders**	**Alcohol use disorders**
Schizophrenia<br>Unipolar depressive disorders<br>• Major depressive disorders<br>• Dysthymia<br>Bipolar affective disorder<br>Anxiety disorders<br>Eating disorders<br>Pervasive development disorders<br>• Autism<br>• Asperger's syndrome<br>Childhood behavioural disorders<br>• Attention-deficit hyperactivity disorder<br>• Conduct disorder<br>Idiopathic intellectual disability<br>Other mental and behavioural disorders	Opioid use disorders<br>Cocaine use disorders<br>Amphetamine use disorders<br>Cannabis use disorders<br>Other drug use disorders	Alcohol use disorders

Table S4: Summary of pooled prevalence of selected mental and substance use disorders in Sub-Saharan Africa from GBD 2010, by region and adjusted for comorbidity

**Region**	**Central**		**East**		**Southern**		**West**	
**Disorder**	**2010**	**2050**	**2010**	**2050**	**2010**	**2050**	**2010**	**2050**
Schizophrenia	0.27%	0.30%	0.26%	0.29%	0.27%	0.31%	0.27%	0.30%
Bipolar	0.88%	0.87%	0.88%	0.87%	0.88%	0.87%	0.87%	0.86%
Depression	5.00%	5.22%	4.76%	5.02%	4.39%	4.62%	3.67%	3.86%
Alcohol	0.88%	0.89%	0.64%	0.64%	1.44%	1.49%	0.40%	0.40%
Opioids	0.13%	0.12%	0.13%	0.12%	0.19%	0.17%	0.13%	0.12%
Conduct	1.88%	1.90%	1.88%	1.90%	1.88%	1.90%	1.86%	1.86%
ADHD	1.00%	1.03%	1.00%	1.03%	1.03%	1.05%	1.03%	1.05%

Table S5: Treatment coverage targets and care packages (taken from Bruckner et al[1])

Epidemiology & Target population		Inpatient service use / needs								Outpatient service use / needs									
		General hospital inpatient unit (acute care)[2]		Community psychiatric (acute care)[3]		Community residential care (long stay)[4]		Mental hospital (long stay)[5]		Primary health care (treatment)		Primary health care (screening)		Psychosocial treatment		Hospital outpatient care		Day care	
	Treatment coverage target[6]	Service coverage[7]	Utilisation rate (days per year)[8]	Service coverage[7]	Utilisation rate (days per year)[8]	Service coverage[7]	Utilisation rate (days per year)[8]	Service coverage[7]	Utilisation rate (days per year)[8]	Service coverage[7]	Utilisation rate (visits per year)[9]	Service coverage[7]	Utilisation rate (visits per year)[9]	Service coverage[7]	Utilisation rate (visits per year)[9]	Service coverage[7]	Utilisation rate (visits per year)[9]	Service coverage[7]	Utilisation rate (days per year)[9]
Schizophrenia	80%	0%	0	15%	28	2.5%	180	2%	90	30%	6	0%	0	30%	8	50%	12	7.5%	100
Bipolar	80%	0%	0	15%	28	2.5%	180	2%	90	30%	6	0%	0	30%	8	50%	12	7.5%	100
Depression	33%	0%	0	2%	14	0.5%	90	0%	0	30%	7	7%	1	20%	6	20%	7	1%	50
Alcohol dependence	25%	0%	0	2%	5	0%	0	0%	0	0%	0	2%	1	25%	3	10%	2	0%	0
Opioid dependence	50%	10%	8	0%	0	2%	60	0%	0	75%	24	0%	0	25%	3	25%	11	6%	26
Childhood conduct disorder	20%	0%	0	2%	2.7	0%	0	0%	0	100%	6.2	0%	0	0%	0	25%	3	2%	6
Childhood ADHD	20%	0%	0	2%	2.7	0%	0	0%	0	100%	6.2	0%	0	0%	0	25%	3	2%	6

[1] Service delivery models for each disorder were taken directly from Bruckner and colleagues' (2011) *Target mental health service delivery models in low- and middle-income countries for the mental, neurological and substance abuse disorders prioritized by the World Health Organization*, which were based on the World Health Organization's sub-regional cost-effectiveness[2] studies and international needs assessment research in developing countries.

[2] Treatment in a general, non-psychiatric bed in a general hospital (for opioid dependence only).

[3] Treatment in a psychiatric bed in a community or general hospital.

[4] Long stay bed based mental health care in a community residential setting.

[5] Long stay bed based mental health care in a psychiatric hospital.

[6] The percentage of prevalent cases requiring or presenting for any treatment.

[7] The percentage of treated cases needing care in this specific service setting in a year.

[8] The average number of bed days used per case per year for cases treated in this specific service setting.

[9] The average number of consultations or sessions per case per year for cases treated in this specific service setting.

Table S6: Staffing ratios

Human resource category	Staffing proportion by health care setting					No. of staff per 25 bed inpatient ward
	**Primary care**	**Ancillary care[1]**	**Outpatient care**	**Day Care**	**Inpatient care**	
Psychiatrist	2%	5%	10%	5%	10%	1
Other physician / doctor	15%	5%	15%	5%	10%	0.5
Nurse	50%	30%	30%	50%	50%	4
Psychologist	3%	5%	10%	10%	10%	0.5
Other psychosocial workers	10%	25%	20%	20%	10%	0.5
Other providers / workers	20%	30%	15%	10%	10%	1
Total	100%	100%	100%	100%	100%	7.5

Source: Dan Chisholm personal correspondence ("The default distribution in the mhGAP costing tool")[3]

[1] Staffing ratios for the ancillary care category from the mhGAP costing tool were applied to care packages for psychosocial treatment from Bruckner et al[2] (as per Table S3).

Key algorithms:

- Target population = total population x disorder prevalence (%) x treatment coverage target (%)
- Number of bed days (inpatients) or sessions (outpatients) per year= target population x % of cases using services x average rate of use (days or sessions per year)
- The number of FTE staff required to deliver outpatient mental health care was calculated as: number of sessions per year / number of consultations per day / 240 working days per year (from mhGAP) X staffing ratio (from mhGAP)
- The number of FTE staff required to deliver inpatient/residential mental health care was calculated as: number of bed-days / 0.85 (85% bed occupancy rate as per Bruckner et al) / 365 service days per annum / 25 beds in unit x number of staff per unit (7.5 per 25 beds as per mhGAP) x staffing ratio (proportion of nurses, doctors, and other professionals in that setting from mhGAP)

Figure S1: Change in burden distribution of communicable diseases due to population ageing and growth

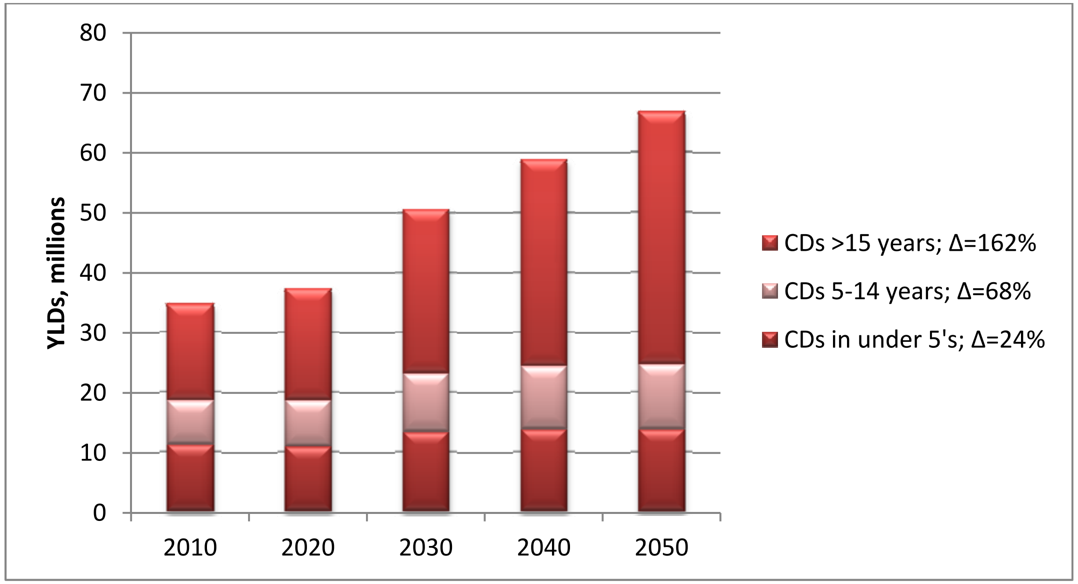

Note: CDs include all communicable, maternal, neonatal and nutritional diseases

Table S7: Estimated increase in mental and substance use disorders YLD, 2010-2050

**Region**	**2010**	**2020**	**2030**	**2040**	**2050**
Sub-Saharan Africa, Central	2,330,646	3,149,658	4,226,315	5,516,692	6,906,692
Sub-Saharan Africa, East	8,373,367	10,988,065	13,968,937	17,051,601	20,009,750
Sub-Saharan Africa, Southern	1,845,833	1,995,444	2,139,705	2,259,494	2,353,826
Sub-Saharan Africa, West	6,757,997	8,744,972	10,980,392	13,285,815	15,462,953
Sub-Saharan Africa, Total	19,316,225	24,893,211	31,320,859	38,087,363	44,690,958

Figure S2: Change in mental and substance use disorders relative to all NCDs

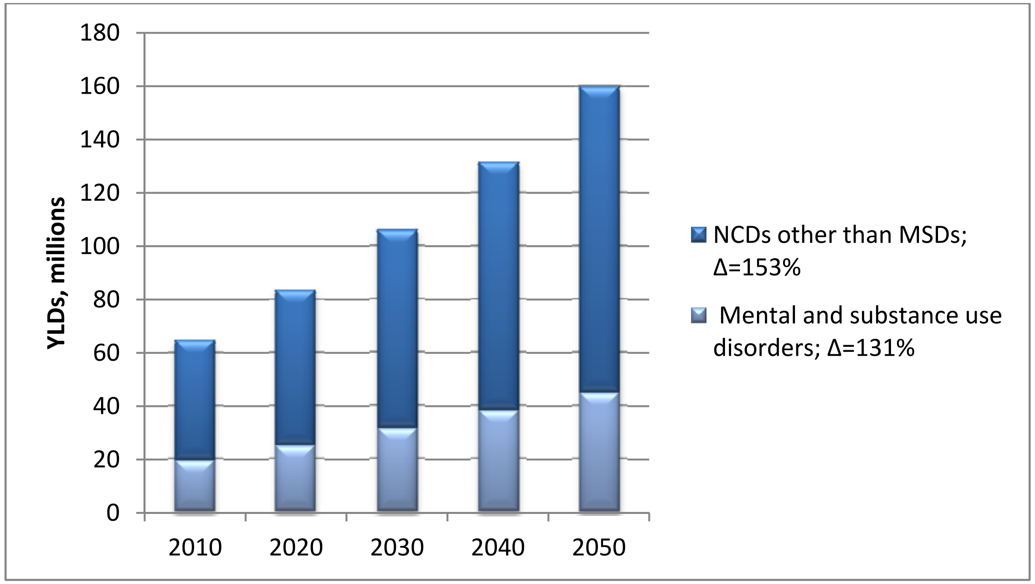

References:

1. Murray CJ, Vos T, Lozano R, Naghavi M, Flaxman AD, et al. (2012) Disability-adjusted life years (DALYs) for 291 diseases and injuries in 21 regions, 1990–2010: a systematic analysis for the Global Burden of Disease Study 2010. The Lancet 380: 2197-2223.

2. Bruckner TA, Scheffler RM, Shen G, Yoon J, Chisholm D, et al. (2011) The mental health workforce gap in low-and middle-income countries: a needs-based approach. Bulletin of the World Health Organization 89: 184-194.

3. Chisholm D. (2014) The default distribution in the mhGAP costing tool.
